# New insights into the ORF2 capsid protein, a key player of the hepatitis E virus lifecycle

**DOI:** 10.1038/s41598-019-42737-2

**Published:** 2019-04-18

**Authors:** Maliki Ankavay, Claire Montpellier, Ibrahim M. Sayed, Jean-Michel Saliou, Czeslaw Wychowski, Laure Saas, Sandrine Duvet, Cécile-Marie Aliouat-Denis, Rayan Farhat, Valentin de Masson d’Autume, Philip Meuleman, Jean Dubuisson, Laurence Cocquerel

**Affiliations:** 1 Univ. Lille, CNRS, Inserm, CHU Lille, Institut Pasteur de Lille, U1019 - UMR 8204 - CIIL- Center for Infection and Immunity of Lille, F-59000 Lille, France; 2 Laboratory of Liver Infectious Diseases, Department of Clinical Chemistry, Microbiology and Immunology, Ghent University, Ghent, Belgium; 3 Microbiology and Immunology Department, Faculty of Medicine, Assiut University, Assiut, Egypt; 4 Univ. Lille, CNRS, UMR 8576 - UGSF - Unité de Glycobiologie Structurale et Fonctionnelle, F-59000 Lille, France; 5 Present Address: Department of Pathology, School of Medicine, University of California, San Diego, La Jolla, California USA

**Keywords:** Virus-host interactions, Glycosylation

## Abstract

Hepatitis E Virus (HEV) genome encodes three proteins including the ORF2 capsid protein. Recently, we demonstrated that HEV produces three different forms of ORF2: (i) the ORF2i form (infectious ORF2) which is the component of infectious particles, (ii) the secreted ORF2g (glycosylated ORF2) and ORF2c (cleaved ORF2) forms that are not associated with infectious particles, but are the major antigens in HEV-infected patient sera. The ORF2 protein sequence contains three highly conserved potential N-glycosylation sites (N1, N2 and N3). The status and biological relevance of ORF2 N-glycosylation in HEV lifecycle remain to be elucidated. Here, we generated and extensively characterized a series of ORF2 mutants in which the three N-glycosylation sites were mutated individually or in combination. We demonstrated that the ORF2g/c protein is N-glycosylated on N1 and N3 sites but not on the N2 site. We showed that N-glycosylation of ORF2 protein does not play any role in replication and assembly of infectious HEV particles. We found that glycosylated ORF2g/c forms are very stable proteins which are targeted by patient antibodies. We also demonstrated that the ORF2i protein is translocated into the nucleus of infected cells. Hence, our study led to new insights into the molecular mechanisms of ORF2 expression.

## Introduction

Hepatitis E virus (HEV) is the most common cause of acute viral hepatitis worldwide and is an emerging problem in industrialized countries. This virus infects approximately 20 million people every year and is responsible for an estimated 3.4 million symptomatic cases and 70,000 deaths, mainly in developing countries^1^. Although HEV infection is usually asymptomatic and self-resolving, severe forms in pregnant women^2^ and chronic infections in immunocompromised patients^3^ have been described. In addition, HEV infection has been associated with extrahepatic manifestations^3^. HEV strains infecting humans have been classified into 4 main distinct genotypes (gt) belonging to a single serotype^4^. Gt1 and gt2 exclusively infect humans and are spread mainly through contaminated drinking water and represent main causes of waterborne outbreaks of hepatitis in developing countries. In industrialized countries, gt3 and gt4 are zoonotic and mainly infect mammals, where pigs and game are the main reservoirs^5^. The major transmission routes of gt3 and gt4 are direct contact with infected animals, consumption of contaminated food and transfusion of blood products^6^.

HEV is a quasi-enveloped virus^7–9^ containing a linear, single-stranded, positive-sense RNA genome with three open reading frames (ORFs), namely, ORF1, ORF2 and ORF3^10^. ORF1 encodes the ORF1 non-structural polyprotein that is essential for viral replication^11^. ORF2 encodes the ORF2 viral capsid protein and ORF3 encodes a small phosphoprotein that is involved in virion morphogenesis and egress^12^.

The ORF2 protein sequence contains 660 amino acids (aa) and displays a signal peptide at its N-terminus and three highly conserved potential N-glycosylation sites represented by the sequon Asn-X-Ser/Thr (N-X-S/T)^13–15^. The ORF2 protein has been largely studied and is the most characterized HEV viral protein^12^. However, the status and biological relevance of ORF2 N-glycosylation is still unclear. Previous attempts to characterize ORF2 N-glycosylation sites were either based on over-expression of recombinant ORF2 protein^15–18^ or by using a gt1 strain which does not robustly replicate in cell culture^14^. By combining the highly replicative and cell culture-selected gt3 p6 strain^19^ and a highly transfectable subclone of PLC/PRF/5 cells (PLC3 cells), we recently developed an efficient HEV cell culture system and demonstrated for the first time that HEV infection leads to the secretion of at least 3 forms of the ORF2 capsid protein: infectious ORF2 (ORF2i), glycosylated ORF2 (ORF2g), and cleaved ORF2 (ORF2c)^20^. We identified the precise sequence of the ORF2i and ORF2g proteins. The ORF2i protein is the structural component of infectious particles. It is not glycosylated and is likely derived from the assembly of the intracellular ORF2 (ORF2intra) form present in the cytosolic compartment. In contrast, ORF2g and ORF2c proteins are secreted in large amounts in culture supernatant and infected patient sera, sialylated, N- and O-glycosylated but are not associated with infectious virions^20^. The identification of these ORF2 forms and post-translational modifications suggest the existence of different pathways for the production of HEV capsid protein, either by differential addressing of ORF2 protein into the secretory pathway^20^ or by a differential translation process, as recently suggested^21^. Importantly, ORF2g and ORF2c proteins are the most abundant antigens detected in patient sera^20^. In addition, it has been very recently shown that these proteins might inhibit antibody-mediated neutralization^21^. Whether ORF2g and ORF2c proteins play a specific role in the HEV lifecycle therefore needs to be elucidated.

In the present study, we took advantage of our HEV cell culture system, in which ORF2 proteins are robustly expressed, to analyze the significance of N-glycosylation of the ORF2 protein in the HEV lifecycle. Using site-directed mutagenesis of the full-length infectious p6 clone, we constructed a series of ORF2 mutants in which the three potential N-glycosylation sites (^137^NLS, N1; ^310^NLT, N2; ^562^NTT, N3) were mutated individually or in combination. We performed an extensive characterization of these mutants by analyzing their subcellular localization, expression, oligomerization, stability and recognition by antibodies. We also studied the impact of mutations on assembly, density and infectivity of HEV particles. In addition to analyzing the status and biological relevance of ORF2 N-glycosylation in the HEV lifecycle, we obtained new insights into the molecular mechanisms of ORF2 biology.

## Results

**Figure 1:**
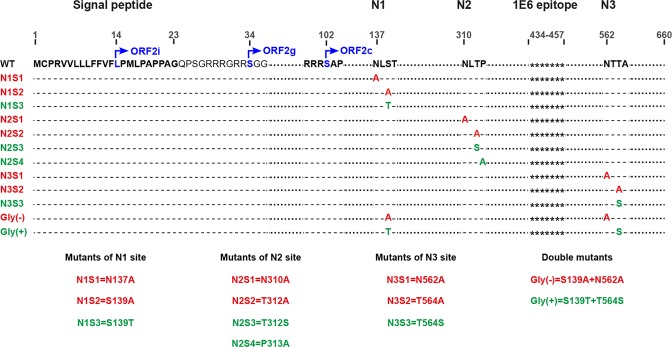
Schematic representation of wild type and mutant ORF2 protein sequences. HEV ORF2 protein is a 660 amino acid protein. The first 23 amino acids (aa) corresponding to the signal peptide are in bold. Positions of the first aa of ORF2i, ORF2g and ORF2c proteins are indicated. The three potential N-glycosylation sites are in bold (N1, N2 and N3). For each mutant, the introduced mutation(s) is/are shown. Mutations that inhibit N-glycosylation are in red whereas mutations that do not inhibit N-glycosylation are in green. The stars (*******) represent the epitope of the 1E6 anti-ORF2 antibody.

The ORF2 protein sequence displays a signal peptide sequence at its N-terminus and three potential N-glycosylation sites, ^137^NLS (N1), ^310^NLT (N2), and ^562^NTT (N3) (Fig. 1). Previously, we demonstrated that the first aa of infectious particle-associated ORF2i and ORF2g proteins are L^14^ and S^34^, respectively^20^. Here, by using the same mass spectrometry approach, we found that the first aa of ORF2c protein corresponds to S^102^ (Figs 1 and S1), indicating that ORF2c protein is likely a cleavage product of the ORF2g protein. The precise sequence of the ORF2intra protein needs to be identified.

### Generation of HEV p6 genomes expressing mutations of ORF2 N-glycosylation sites

Using site-directed mutagenesis of the full-length infectious p6 clone, we constructed a series of ORF2 mutants in which N1, N2 and N3 sites were mutated individually (N1, N2 and N3 mutants) or in combination (Gly(−) and Gly(+) mutants) (Fig. 1). For each single mutant, three substitutions of the N-X-S/T sequon were generated: N-to-A (S1), S/T-to-A (S2) and S-to-T/T-to-S (S3). S1 and S2 substitutions were made to prevent N-glycosylation (Fig. 1, highlighted in red), whereas S3 substitution does not affect N-glycan addition (Fig. 1, highlighted in green). A fourth substitution (S4) was done for the N2 site in which a Proline (P) residue downstream of the sequon was replaced by an Alanine (P313A). In Gly(−) and Gly(+) mutants, two combinations of mutations affecting (N1S2/N3S1) or not (N1S3/N3S3) the N-glycan addition on N1 and N3 glycosylation sites were introduced, respectively (Fig. 1). Capped RNAs transcripts were generated and delivered into PLC3 cells by electroporation.

### Expression and subcellular localization of mutant ORF2 proteins

**Figure 2:**
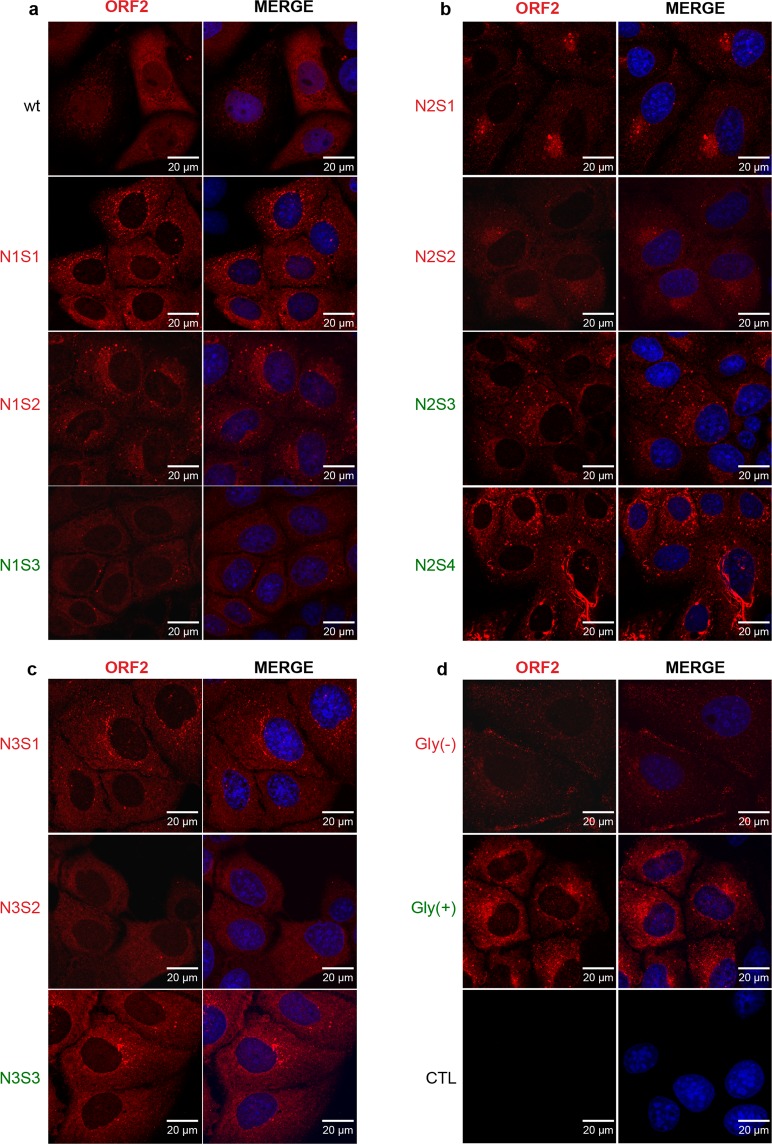
Subcellular distribution of wt and mutant ORF2 proteins. PLC3 cells were electroporated with wt and mt HEV RNAs and grown on coverslips. 3 days post-electroporation, cells were fixed and ORF2 protein was stained using the 1E6 antibody (in red). Nuclei were stained with DAPI (in blue). Cells were analyzed by confocal microscopy (magnification x40). Representative acquisitions of two independent experiments are presented. (**a**) Cells expressing wild type (wt) and N1S1 (N137A); N1S2 (S139A); N1S3 (S139T) mutants. (**b**) Cells expressing N2S1 (N310A); N2S2 (T312A); N2S3 (T312S) and N2S4 (P313A) mutants. (**c**) Cells expressing N3S1 (N562A); N3S2 (T564A); N3S3 (T564S) mutants. (**d**) Cells expressing Gly(−) (N1S2+N3S1) and Gly (+) (N1S3 + N3S3) double mutants and non-transfected PLC3 cells (CTL).

We first evaluated the expression and subcellular localization of mutant ORF2 proteins by indirect immunofluorescence. PLC3 cells electroporated with wild type and mutants of HEV RNAs (wt and mt PLC3/HEV cells) were fixed at 3 days post-electroporation (d.p.e.) and processed for ORF2 staining. For all constructs, over 90% of cells were ORF2-positive indicating that robust transfection and expression of viral genome occurred in wt and mt PLC3/HEV cells (data not shown). As shown in Fig. 2a, the wt ORF2 protein displayed mostly a cytoplasmic localization but likely also a nuclear localization, as recently described^22^. Although mutant ORF2 proteins were globally expressed as the wt ORF2 protein, N1 mutants showed a slightly more perinuclear localization (Fig. 2a), N2 mutants showed a concentrated staining in a spot close to the nucleus (Fig. 2b) whereas N3 mutants showed a subcellular localization similar to that of wt ORF2 proteins (Fig. 2c). Gly(−) and Gly(+) double mutants displayed a subcellular localization similar to that of N1 and N3 mutants, respectively (Fig. 2d). Double labeling with anti-ORF2 and ER-specific anti-calnexin MAbs or Golgi-specific anti-GM130 MAbs failed to reveal co-localization of ORF2 proteins with these compartment markers (data not shown), as previously observed for the gt1 ORF2 protein^14^. Nuclear localization of wt and mt ORF2 proteins was further characterized, as described below.

### The ORF2 protein is N-glycosylated on N1 and N3 sites but not on the N2 site

**Figure 3:**
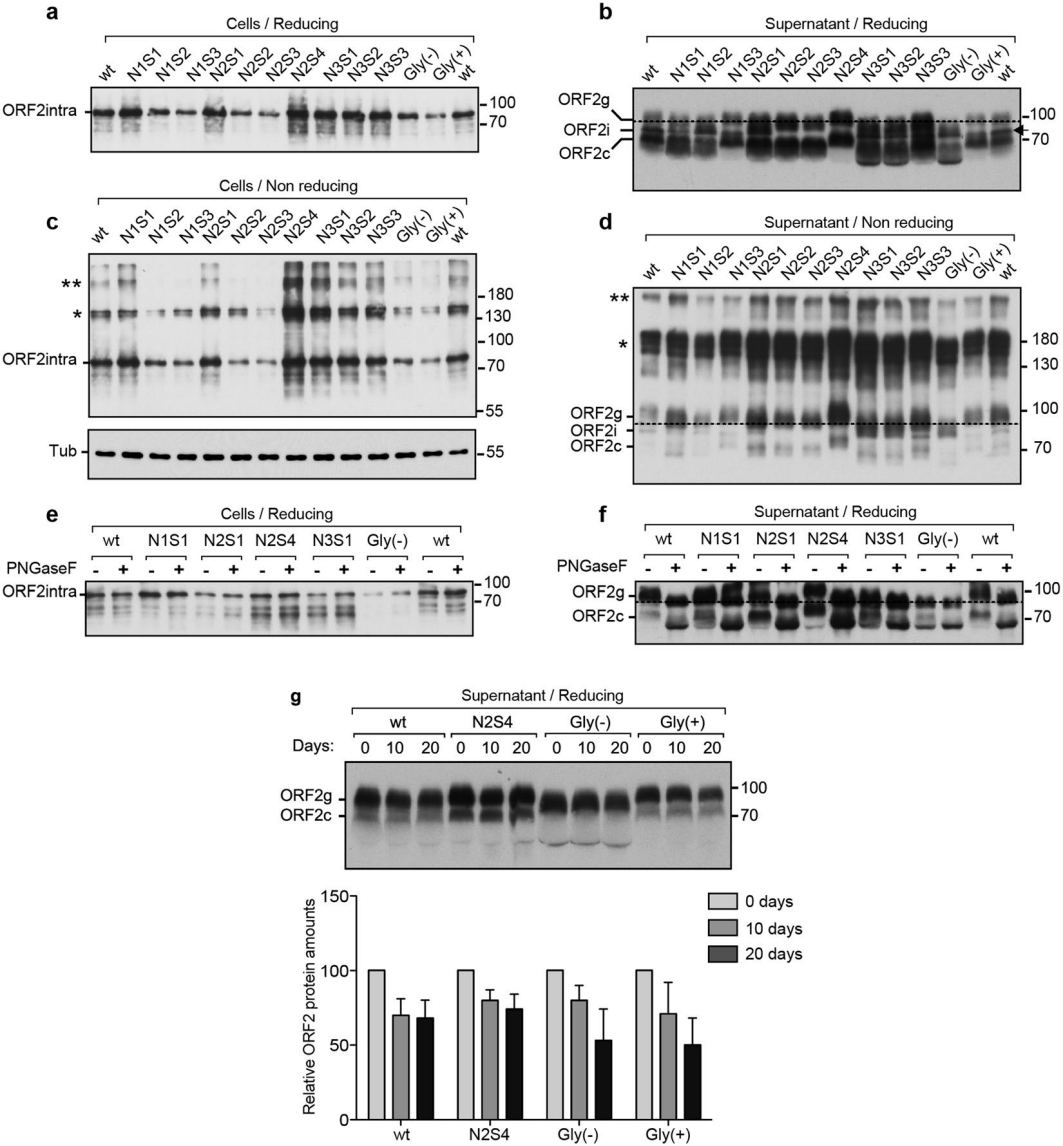
Characterization of wt and mutant ORF2 proteins by western blot (WB). Supernatants and lysates of wt and mt PLC3/HEV cells were collected at 10 days post-electroporation, normalized by protein quantification assay, and ORF2 protein was detected by WB using the 1E6 antibody. The representative results of two independent experiments are presented. Detection of ORF2intra protein in cell lysates in reducing conditions (**a**) and in non-reducing conditions (**c**). Tubulin (Tub) was also detected to control protein loading. Detection of ORF2 protein in supernatants in reducing conditions (**b**) and in non-reducing conditions (**d**). In (**b**), the arrow indicates the ORF2i protein. Wt and mt PLC3/HEV cell lysates (**e**) and supernatants (**f**) were digested (+) or not (−) with Peptide-N-Glycosidase F (PNGaseF). (**g**) Supernatants of wt and mt PLC3/HEV cells were incubated for indicated times at 37 °C. Relative ORF2 protein amounts were measured by densitometry of shorter exposures. Values were adjusted to 100% for time 0 day. Results are from three independent experiments. In (**b**), (**d**) and (**f**), the dashed line was used to evaluate the shift in migration profile. For clarity and conciseness concerns, blots were cropped. Full-length blots are presented in Supplementary Fig. 3.

ORF2 protein expressions in supernatants and lysates of wt and mt PLC3/HEV cells were characterized by western blotting (WB) by using the 1E6 anti-ORF2 monoclonal antibody that specifically recognizes the different forms of ORF2 protein (^20^ and Fig. S2). A major band corresponding to the ORF2intra protein was detected in all cell lysates (Fig. 3a), as previously described^20^. Stainings observed in Fig. 2 thus corresponded mainly to the subcellular localization of ORF2intra proteins. We observed no shift of migration between wt and mutant proteins, even for the constructs carrying mutations that inhibit N-glycosylation. These results confirm that the ORF2intra protein is not N-glycosylated. In cell supernatants, two intense bands corresponding to the ORF2g and ORF2c forms were identified (Fig. 3b), as previously described^20^. A very faint band corresponding to the ORF2i protein was also detected in some samples i.e. right wt and Gly(+) (Fig. 3b, indicated by an arrow). Interestingly, we observed a shift of migration of ORF2g and ORF2c proteins for the mutants that inhibit N-glycosylation on N1 or N3 sites or both (N1S1, N1S2, N3S1, N3S2 and Gly(−)), as compared to wt protein (Fig. 3b,d). In contrast, no shift of migration was observed for proteins carrying mutations that do not affect glycosylation of N1 and N3 sites (N1S3, N3S3 and Gly(+)). These results indicate that both N1 and N3 sites of the ORF2 protein are likely occupied by N-glycans. Analyses of N2S1, N2S2 and N2S3 mutants showed that their ORF2g and ORF2c proteins behaved similarly irrespective of the introduced mutation (affecting or not glycosylation) that prevented us to conclude about the N-glycosylation status of the N2 site. However, since it has been demonstrated that a Proline residue right downstream of the N-X-S/T sequon constitutes an unfavorable context for N-linked glycan modification^23^, we generated an additional mutant of the N2 site in which we replaced the Proline^313^ by an Alanine residue (N2S4) (Fig. 1). Interestingly, N2S4 ORF2g and ORF2c proteins displayed a higher apparent molecular weight than the wt forms (Fig. 3b), indicating that the N2S4 mutation likely led to the addition of a supplementary N-glycan on ORF2 proteins. Together, these results indicated that the N2 site is likely not N-glycosylated in the context of wt ORF2 proteins.

To further characterize the sites of ORF2 protein that are N-glycosylated, representative mutants were selected (N1S1, N2S1, N2S4, N3S1 and Gly(−)) and further characterized. Supernatants and lysates of wt and mt PLC3/HEV cells were denatured and digested with Peptide-N-Glycosidase F (PNGaseF), a glycosidase that cleaves between the innermost N-acetyl glucosamine and asparagine residues of N-glycoproteins. As shown in Fig. 3e, wt and mt ORF2intra proteins expressed from cell lysates were resistant to glycosidase digestion, strengthening the fact that ORF2intra protein in cell lysates is not N-glycosylated. In contrast, we observed a shift of migration between untreated (−) and treated (+) condition for the secreted wt and mutants N1S1, N2S1, N2S4 and N3S1 (Fig. 3f). Importantly, no shift was observed for the Gly(−) double mutant that inhibit N-glycosylation on N1 and N3 sites, confirming that the N2 site is not occupied by N-glycans. In addition for the N2S4 mutant, we observed a higher migration shift between PNGaseF untreated (−) and treated (+) ORF2g and ORF2c proteins, as compared to wt (Fig. 3f).

To further confirm which sites of N-glycosylation are occupied by glycans on ORF2 protein, we next performed mass spectrometry analyses. ORF2g/ORF2c proteins immunoprecipitated with an anti-ORF2 antibody (4B2) were treated or left untreated with PNGaseF. Proteins were resolved by SDS-PAGE and Colloïdal blue-stained bands corresponding to ORF2g and ORF2c proteins in WB (data not shown) were digested in-gel with trypsin (Tryp) or the endoproteinase AspN and then analyzed by nano scale liquid chromatography coupled to tandem mass spectrometry. Detected peptides were compared to the theorical peptide sequences of non N-glycosylated or PNGaseF-deglycosylated ORF2 protein digested with Tryp or AspN. As shown in Table 1, when ORF2g/c proteins were not treated with PNGaseF, no peptides covering N1 and N3 sites were detected after digestion with Tryp or AspN. In contrast, upon treatment with PNGaseF, peptides covering N1 and N3 sites were detected after digestion with Tryp or AspN, indicating that N1 and N3 sites are most likely occupied by N-glycans. Interestingly, peptides covering the N2 site were detected in both treated and untreated samples digested with Tryp or AspN, indicating that the probability that the site N2 is occupied by N-glycans is very low (Table 1).

**Table 1:** Mass spectrometry analyses of ORF2 N-glycosylation sites.

	Theorical peptide sequences of non N-glycosylated or PNGaseF-deglycosylated ORF2 protein digested with Trypsin (Tryp) or AspN			Number of MSMS spectrum for ORF2g/ORF2c		Normalized total area of peptide peak for ORF2g/ORF2c		N-glycosylation probability
				Non treated	Treated by PNGaseF	Non treated	Treated by PNGaseF	
**Site N1**	Tryp	Non N-glycosylated	DVDSRGAIRRQYNLSTSPLTSSVASGTNLVLYAAPLNPLLPLQDGTNTHIMATEASNYAQYR	ND^a^	ND	ND	ND	High
		Deglycosylated by PNGaseF^b^	DVDSRGAIRRQYDLSTSPLTSSVASGTNLVLYAAPLNPLLPLQDGTNTHIMATEASNYAQYR	ND	ND	ND	ND	
	AspN	Non N-glycosylated	DVDSRGAIRRQYNLSTSPLTSSVASGTNLVLYAAPLNPLLPLQ	ND	ND	ND	ND	
		Deglycosylated by PNGaseF^c^	DVDSRGAIRRQY	ND	2/2	0.0/0.0%	0.5/1.2%	
**Site N2**	Tryp	Non N-glycosylated	DFALELEFRNLTPGNTNT	9/6	9/8	6.8/4.6%	5.7/4.1%	Low
		Deglycosylated by PNGaseF	DFALELEFRDLTPGNTNT	ND	ND	ND	ND	
	AspN	Non N-glycosylated	DFALELEFRNLTPGNTNTRVSRYTSTAQHRLRRGA	ND	ND	ND	ND	
		Deglycosylated by PNGaseF	DFALELEFR	2/2	6/6	0.1/0.2%	2.4/2.3%	
**Site N3**	Tryp	Non N-glycosylated	EAGTTRAGYPYNYNTTASDQILIENAAGHR	ND	ND	ND	ND	High
		Deglycosylated by PNGaseF	EAGTTRAGYPYNYDTTASDQILIENAAGHR	ND	13/8	0.1/0.0%	5.6/7.4%	
	AspN	Non N-glycosylated	EAGTTRAGYPYNYNTTAS	ND	ND	ND	ND	
		Deglycosylated by PNGaseF	EAGTTRAGYPYNY	ND	2/2	0.0/0.0%	1.1/0.8%	

^a^Not detected.

^b^Upon PNGaseF treatment, the asparagine (N) residues from which the glycans have been removed are deaminated to aspartic acid (D) residues.

^c^Deamination of glycosylated N residues to D residues by PNGaseF treatment generates cleavage sites for AspN.

Taken together, our results demonstrated that the ORF2 protein is N-glycosylated on N1 (^137^NLS) and N3 (^562^NTT) sites but not on the N2 (^310^NLT) site.

### Impact of mutations of N-glycosylation sites on the oligomerization and stability of ORF2

We next assessed the impact of mutations of N-glycosylation sites on ORF2 oligomerization by analyzing supernatants and lysates of wt and mt PLC3/HEV cells prepared in non-reducing conditions. Oligomers of ORF2intra and glycosylated ORF2 proteins were clearly detected (Fig. 3c,d, indicated by asterisks). Taking into account the differences in individual protein expression levels (Fig. 3a), no significant difference in the oligomerization levels of ORF2 proteins was observed between wt and mutants.

We next analyzed the significance of ORF2 N-glycosylation for protein stability. For this purpose, supernatants of wt and mt PLC3/HEV cells were kept during 0, 10 and 20 days at 37 °C and analyzed by WB (Fig. 3g). Surprisingly, even after 20 days of incubation, ORF2g and ORF2c proteins were still readily detected, indicating that these proteins are very stable and poorly degraded in culture medium. Quantification by densitometry of ORF2 levels in wt and N2S4 supernatants, showed that only 30–35% of proteins were degraded after 20 days. In contrast, 50% of Gly(−) and Gly(+) proteins were degraded after 20 days, indicating that these mutants are less stable in culture medium but in a glycosylation-independent manner.

### Mutations of N-glycosylation sites do not modify ORF2 antibody recognition

**Figure 4:**
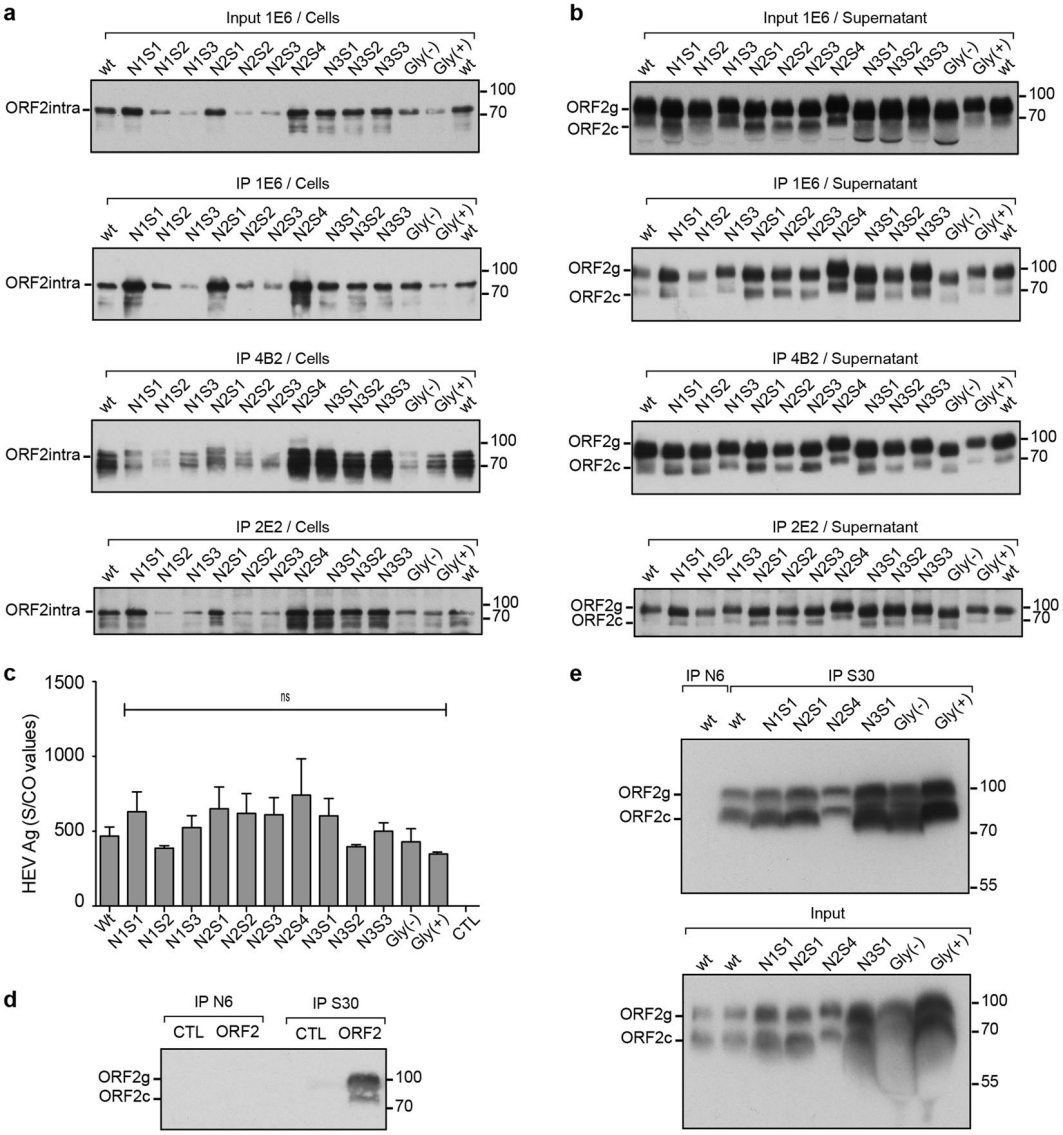
Impact of mutations of ORF2 protein N-glycosylation sites on antibody recognition. (**a**,**b**) Supernatants and lysates of wt and mt PLC3/HEV cells were collected at 10 days post-electroporation. Proteins in cell lysates and supernatants were normalized by protein quantification assay on cell lysates. ORF2 proteins were immunoprecipated (IP) by using the 1E6 linear anti-ORF2 antibody or the 4B2 and 2E2 conformational anti-ORF2 antibodies, as indicated. Input of ORF2 proteins used for immunoprecipitations are shown. ORF2 proteins were detected by WB using 1E6 antibody. (**c**) Detection of HEV-Ag in supernatants using the Wantaï HEV-Ag ELISA^Plus^ kit. Results are presented as signal to cut-off ratios (S/CO). (**d**) A serum from a non-infected patient (N6) and a serum from a patient who has cleared his HEV infection (S30) were both incubated with protein A-agarose beads and then with ORF2g/ORF2c proteins (ORF2) or PBS (CTL). ORF2 proteins were next detected by WB using 1E6 antibody. ORF2g/ORF2c proteins were isolated on iodixanol cushions (**e**) (Top) ORF2g/c proteins isolated on iodixanol cushions from supernatants of wt and mt PLC3/HEV cells were immunoprecipitated with S30-immobilized beads. ORF2g/c proteins from supernatant of wt PLC3/HEV cells immunoprecipitated with N6-immobilized beads were used as a control. (Bottom) Input of ORF2g/ORF2c proteins used for immunoprecipitations are shown. Representative results of two independent experiments are shown. For clarity and conciseness concerns, blots were cropped. Full-length blots are presented in Supplementary Fig. 4.

To determine the impact of mutations of N-glycosylation sites on the anti-ORF2 antibody recognition, ORF2 proteins in cell lysates and supernatants of wt and mt PLC3/HEV cells were immunoprecipitated with different antibodies and analyzed by WB (Fig. 4). We used the linear 1E6 antibody of which the epitope is located at amino acids 434–457, and two conformational antibodies having neutralizing properties (4B2 and 2E2)^24^. Although some detection differences were observed for several mutants, likely reflecting differences in individual protein expression levels (Fig. 4a, input), mutant ORF2intra proteins were recognized by the three antibodies (Fig. 4a). It has to be noted that some mutants displayed additional ORF2 products that need to be further characterized. The supernatants were standardized according to the ORF2i expression levels and then immunoprecipitated. ORF2g and ORF2c proteins were equally immunoprecipitated by the three antibodies (Fig. 4b). We also quantified levels of secreted ORF2 proteins with the Wantaï HEV-antigen ELISA^Plus^ assay that has been recently marketed for HEV diagnosis and that works with monoclonal and polyclonal antibodies. As shown in Fig. 4c, no significant differences were observed in protein detection between wt and mt ORF2 proteins.

We next sought to determine whether mutations of N-glycosylation sites affect recognition by patient antibodies. For this purpose, we used a serum from a patient who had cleared HEV infection (serum S30) and a serum from a non-infected patient (serum N6). Sera were incubated with protein A-agarose beads and then with ORF2g/ORF2c proteins or PBS (CTL) (Fig. 4d). ORF2g/ORF2c proteins were isolated on iodixanol cushions, as previously described^20^. ORF2g and ORF2c proteins were specifically immunoprecipitated by immunoglobulins raised in the S30 patient serum whereas no proteins were precipitated by the N6 negative serum. As shown in Fig. 4e, wt and mt ORF2g/ORF2c proteins were all recognized by antibodies from the S30 patient serum.

Taken together, these results demonstrated that mutations of ORF2 N-glycosylation sites do not modify the recognition of ORF2 protein by linear, conformational and patient antibodies. Thus, these mutations induced no major changes in ORF2 protein folding and N-glycosylation of ORF2 protein did not play a major role in antibody recognition. Importantly, our results showed that ORF2g and ORF2c proteins were highly recognized by patient antibodies.

### The ORF2 protein translocates into the nucleus of infected cells independently of its N-glycosylation status

**Figure 5:**
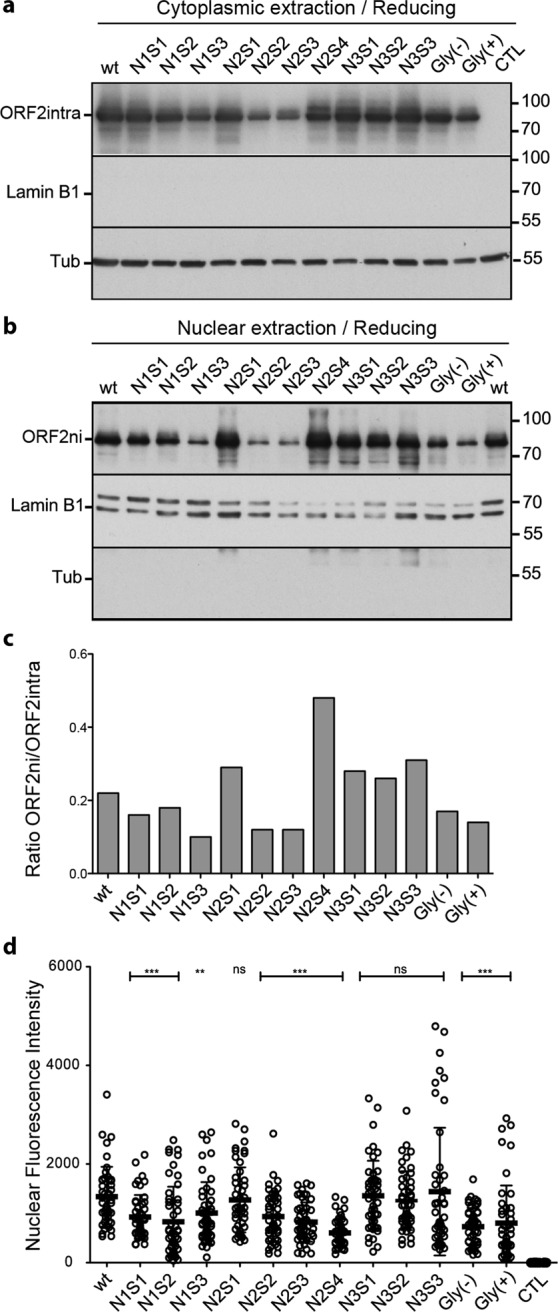
Impact of mutations of N-glycosylation sites on ORF2 protein nuclear localization. Cytoplasmic (**a**) and nuclear (**b**) extracts from wt and mt PLC3/HEV cells were prepared with the NE-PER Nuclear and Cytoplasmic extraction Kit. ORF2intra and ORF2ni (nuclear ORF2intra) proteins were detected by WB. LaminB1 and tubulin (Tub) were also detected to evaluate the quality of extractions. Representative results of two independent experiments are shown. (**c**) Intensity of ORF2 bands in cytoplasmic and nuclear fractionations were quantified by densitometry from shorter exposures of blots. ORF2ni/ORF2intra ratios were next calculated. It has to be noted that since four times more of nuclear extracts were loaded on gels, as compared to cytosolic extracts, ORF2ni/ORF2intra ratios were divided by four. (**d**) wt and mt PLC3/HEV cells were fixed at 3 d.p.e., and ORF2 protein was stained by using the 1E6 antibody. Cells (n = 50) were analysed by LSM 800 confocal laser-scanning (Zeiss) using x40 oil immersion lens. The nuclear fluorescence intensity of ORF2 protein was determined using the ImageJ software. For clarity and conciseness concerns, blots were cropped. Full-length blots are presented in Supplementary Fig. 5.

Since a recent study^22^ and our confocal microscopy analyses (Fig. 2) suggested that, in addition to its cytoplasmic localization, the ORF2 protein might also be translocated into the nucleus of infected cells, we next further characterized this process by WB and immunofluorescence. We prepared cytoplasmic and nuclear extracts from wt and mt PLC3/HEV cells. ORF2 proteins were detected by WB. Cytoplasmic-specific anti-tubulin and nuclear envelope-specific anti-Lamin-B1 antibodies were used to control the quality of extractions. As expected, the ORF2intra protein was detected in cytoplasmic extracts of wt and mt PLC3/HEV cells (Fig. 5a). Interestingly, wt and mutant ORF2 proteins were also detected in nuclear extracts (Fig. 5b). We named this protein ORF2ni for nuclear ORF2intra. Among mutants of N-glycosylation, some differences in the ORF2ni detection (Fig. 5b) and on the ORF2ni/ORF2intra ratio (Fig. 5c) were observed. N1S3, N2S2, N2S3, Gly(−) and Gly(+) mutants showed reduced ORF2ni amounts whereas nuclear translocation was likely not affected for the other mutants, as compared to the wt protein. In order to quantify the effect of mutations of N-glycosylation sites on nuclear translocation, nuclear fluorescence intensity of ORF2 protein was measured on 50 cells for each mutant with the ImageJ software. A significant reduction of nuclear translocation was observed for all mutants excepted for the N2S1 mutant and the three mutants of the N3 site (Fig. 5d**)**. However, we did not observe any correlation between the status of N-glycosylation of ORF2 and its nuclear localization.

Altogether, our results demonstrated that, during HEV lifecycle, ORF2 capsid protein was translocated into the nucleus of infected cells. This nuclear localization was not closely related to the ORF2 N-glycosylation.

### Impact of mutations of ORF2 N-glycosylation sites on viral assembly and infectivity

**Figure 6:**
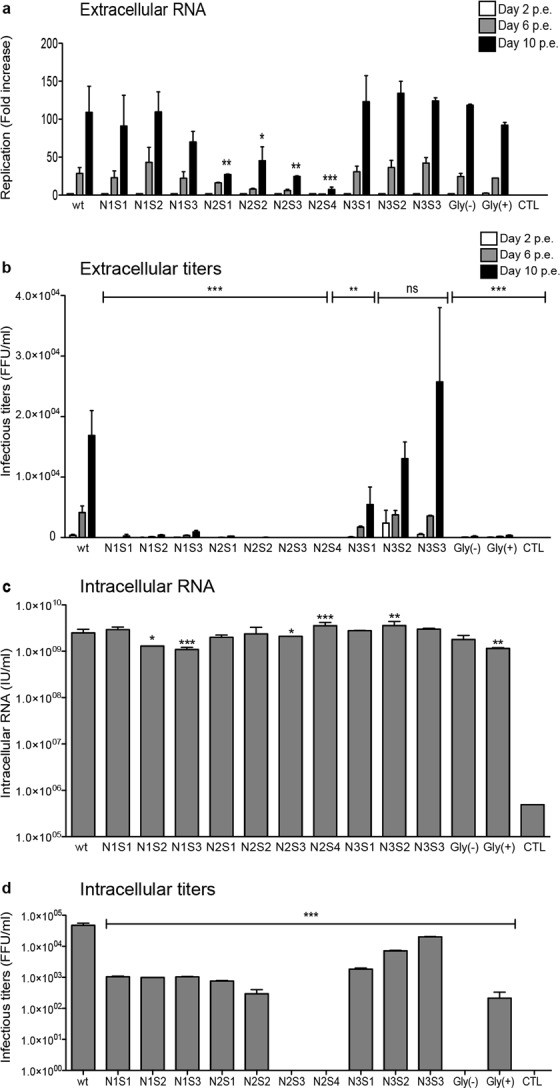
Impact of mutations of ORF2 protein N-glycosylation sites on viral assembly and infectivity. (**a**) The level of HEV RNAs in the supernatants of wt and mt PLC3/HEV cells collected at 2, 6 and 10 d.p.e. was measured by RT-qPCR (IU/ml). For each condition, values are presented as fold increase compared to RNA level measured at 2 d.p.e. Presented data are the mean of two independent experiments performed in duplicate (**b**) Naïve Huh.7.5 cells were infected with serial dilutions of supernatants collected at 2, 6 and 10 d.p.e. At 3 days post-infection, cells were fixed and ORF2 protein was detected by immunofluorescence. Focus forming Units (FFU) were determined and the results are presented as FFU/mL. Presented data are the mean of two independent experiments performed in triplicate. (**c**) Intracellular viral particles were extracted at 10 d.p.e. and the level of HEV RNAs was measured by RT-qPCR (IU/ml). Presented data are the mean of two independent experiments performed in duplicate. (**d**) An aliquot of intracellular viral particles was used to infect naïve Huh. 7.5 cells and the expression of ORF2 protein was analyzed by immunofluorescence. The Focus forming Unit (FFU) was determined and the results are presented as FFU/mL. The shown data are the mean of two independent experiments performed in triplicate.

Next, we analyzed the impact of mutations of ORF2 N-glycosylation sites on viral RNA production and infectivity. Supernatants of wt and mt PLC3/HEV cells were collected at 2, 6 and 10 d.p.e. and then processed for RNA level quantification by RT-qPCR (Fig. 6a) and determination of extracellular infectious titers (Fig. 6b). To specifically quantify capsid-protected RNA genomes, extractions and quantifications of extracellular RNA were performed after treatment of supernatants with RNase. Quantification of extracellular RNA genomes and calculation of fold increase between 2, 6 and 10 d.p.e., which were in line with HEV RNA replication and secretion of capsid-protected RNA genomes (HEV particles), showed that mutants of the N2 site led to a reduction of RNA replication and/or secretion/assembly of capsid-protected RNA genomes (Fig. 6a). In contrast, extracellular RNA levels of N1, N3 and double mutants were similar to wt, indicating that these mutations did not affect HEV RNA replication and secretion of capsid-protected RNA genomes. To determine viral infectivity, Huh-7.5 cells were infected with serial dilutions of supernatants and processed for ORF2 staining at 3 days post-infection. Viral titers were determined by quantifying focus forming units (FFU/mL) (Fig. 6b). Supernatants of N2 mutants were not infectious. Supernatants of N1 and double mutants displayed reduced infectivity. Although the N3S1 mutant was slightly less infectious, other mutants of the N3 site displayed infectious titers similar to wt strain. These results indicated that mutations of the N1 site inhibited infectivity of viral particles whereas mutations of the N3 site had no major impact on the biogenesis of infectious HEV particles.

In order to precisely define the impact of mutations on HEV RNA replication, we quantified the levels of intracellular viral RNA genomes. Although small significant differences were observed among mutants, intracellular RNA replication was globally not affected by the mutations of ORF2 N-glycosylation sites (Fig. 6c). Finally, we determined the infectivity of intracellular particles produced in wt and mt PLC3/HEV cells (Fig. 6d). All the N1 and N2 mutants showed a reduced infectivity of intracellular particles, indicating that the N1 and N2 mutations were lethal for viral infectivity. The intracellular titers of the N3 mutants were also reduced, as compared to wt strain.

Taken together, our results showed that (i) mutations of N1 glycosylation site inhibited infectivity of HEV particles, (ii) mutations of N2 glycosylation site inhibited infectivity and assembly/secretion of infectious particles, and (iii) mutations of N3 glycosylation site had little or no effect on infectivity of secreted HEV particles. Importantly, whatever the introduced mutation within the same site (affecting or not N-glycosylation) the same phenotype was observed indicating that N-glycosylation of ORF2 protein did not play any role in the assembly and infectivity of HEV particles.

### Impact of mutations of ORF2 protein N-glycosylation sites on particle density

**Figure 7:**
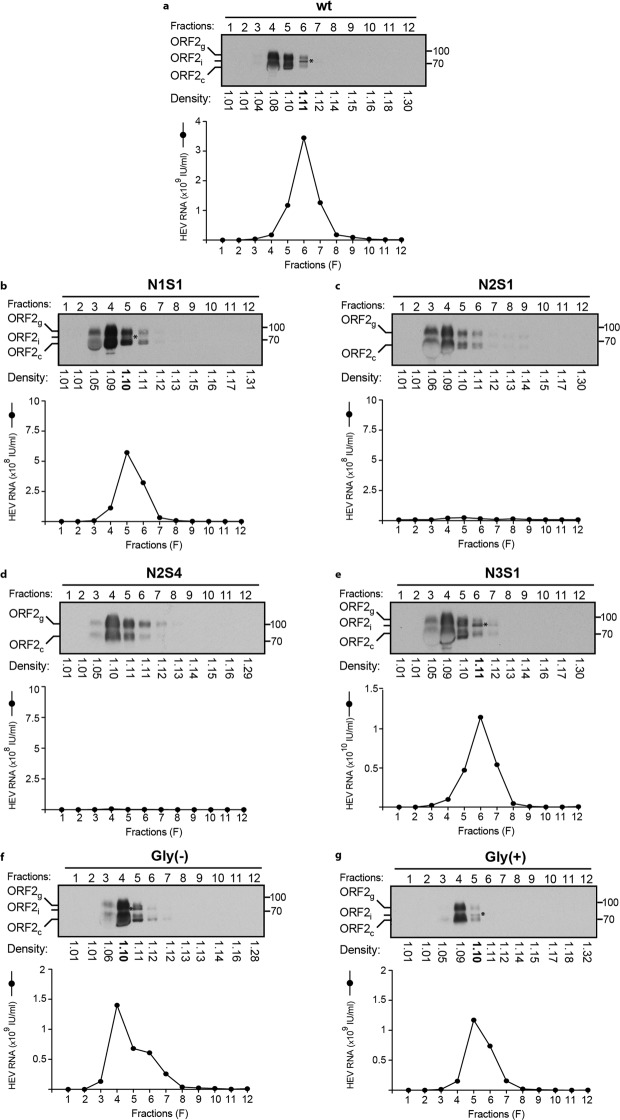
Impact of mutations of ORF2 protein N-glycosylation sites on particle density. Concentrated wt and mt PLC3/HEV cell supernatants were layered on an iodixanol gradient and ultracentrifuged. Twelve fractions were collected and their densities were measured. ORF2 expression was analyzed by WB using 1E6 antibody. Asterisks indicate the ORF2i protein. HEV RNA levels in each fraction were quantified by RT-qPCR. For clarity and conciseness concerns, blots were cropped. Full-length blots are presented in Supplementary Fig. 6.

To further characterize our mutants, we produced large amounts of infectious supernatants by culturing wt and mt PLC3/HEV cells during 12 days. Supernatants were pooled, concentrated 100 times, and fractionated on an iodixanol gradient. ORF2 protein expression, density and RNA levels were determined for each fraction (Fig. 7a–g). As previously, only some representative mutants were analyzed (N1S1, N2S1, N2S4, N3S1, Gly(−) and Gly(+)). ORF2g and ORF2c proteins were mainly detected in fractions 3, 4 and 5 whereas the ORF2i protein was mainly observed (Fig. 7, asterisks) in fractions 5 or 6 of wt (Fig. 7a, lane 6) and mutants that assembled particles: N1S1 (Fig. 7b, lane 5), N3S1 (Fig. 7e, lane 6), Gly(−) (Fig. 7f, lane 5) and Gly(+) (Fig. 7g, lane 5). In accordance with our previous results (Fig. 6), N2S1 and N2S4 mutants displayed no ORF2i and no extracellular RNAs confirming that the mutations of N2 glycosylation site inhibited assembly of HEV particles. In wt and N3S1 gradients, only one major peak of RNA was detected in fraction 6 with a density of 1.11 g/mL. In contrast, N1S1, Gly(−) and Gly(+) displayed a RNA peak in fraction 4 or 5 with a density of 1.10 g/mL, indicating that non-infectious mutants might display a slightly lower density, as compared to infectious particles.

## Discussion

We recently demonstrated that HEV secretes three different forms of its ORF2 capsid protein during its lifecycle^20^. The ORF2i form (∼80 kDa) is the component of infectious particles that is neither N-glycosylated or nor O-glycosylated. In contrast, the ORF2g (∼90 kDa) and ORF2c (∼75 kDa) forms are N-glycosylated, O-glycosylated and sialylated. They are massively secreted but are not associated with infectious material. Recently, Yin *et al*. confirmed our observations that ORF2g and ORF2i forms (named “ORF2^S”^ and “ORF2^C”^ in their study) are produced during HEV lifecycle and they showed that these proteins might be translated from different start codons^21^. However, they did not identify the ORF2c form, likely due to their lower protein expression level, as compared to our system. In the present study, we identified the precise N-terminal sequence of the ORF2c form (S^102^). It is likely a cleavage product of the ORF2g protein and the presence of RRR residues upstream of the ORF2c N-terminus suggests that a furin-like protease might be involved.

Importantly, ORF2g/c proteins do not form particulate material but they are the major viral antigens present in the serum of HEV-infected patients. HEV may produce ORF2g/c proteins as immunological bait. Indeed, it has been very recently shown that secreted ORF2 might inhibit antibody-mediated neutralization^21^. In addition, since these proteins are likely extremely stable in infected patients^25^, they might represent markers of the evolution of hepatitis E infection. It is therefore essential to determine the molecular and cellular mechanisms leading to the biogenesis of the different forms of ORF2 protein.

By over-expression of recombinant gt1 ORF2 protein, it has been previously shown that this protein is an 88 kDa glycoprotein which is expressed intracellularly as well as on cell surface and has the potential to form non covalent homodimers^16^. It has also been suggested that Asn-310 (N2 site) is the major but not the only site of N-glycan addition^15^. The impact of mutations within highly conserved glycosylation sites of the gt1 Sar55 infectious full-length genome has also been studied^14^. However, in this study the authors only analyzed intracellular ORF2 proteins, which are non-glycosylated proteins^20,21^. They demonstrated that mutation of the first two glycosylation sites prevented virion assembly whereas mutation of the third site affected infectivity of particles. Moreover, they showed that mutations of N-glycosylation sites were lethal due to their effect on protein structure rather than the absence of glycosylation^14^. More recently, a triple mutant of N-glycosylation sites confirmed the presence of N-glycans on secreted ORF2 protein^21^. In our study, by using site-directed mutagenesis of the three N-glycosylation sites, PNGaseF treatment and mass spectrometry analyses of ORF2 protein expressed from our efficient gt3 p6 full-length strain-based cell culture system, we robustly demonstrated that the ORF2 protein is N-glycosylated on N1 (^137^NLS) and N3 (^562^NTT) sites but not on the N2 (^310^NLT) site. The absence of N-glycan addition on N2 site is likely due to the presence of a Proline residue immediately downstream of the ^310^NLT sequon, which has a deleterious effect on N-glycosylation^26^. In accordance with this claim, we showed that the P313A substitution (N2S4 mutant) induced additional ORF2 N-glycosylation. Interestingly, alignment of 293 sequences from gt1 to gt4 HEV strains showed that the P^313^ is highly conserved^27^, suggesting that the absence of N-glycans on ORF2 N2 site is likely a conserved feature among HEV genotypes. Hence, our study is the first exhaustive report on ORF2 N-glycosylation in the physiological context of an infectious clone.

We performed an extensive characterization of ORF2 mutants in which the three potential N-glycosylation sites were mutated individually or in combination. Whatever the introduced mutations, we observed no significant differences in expression, secretion, oligomerization, stability and recognition of the secreted ORF2g and ORF2c proteins by several antibodies. However, we cannot exclude that mutations affect recognition by some other neutralizing antibodies^21^. We showed that ORF2g and ORF2c proteins were very stable and poorly degraded in cell culture, which is consistent with previous *in vivo* observations^25^. Importantly, we showed that ORF2g and ORF2c proteins were the main antigens recognized by antibodies from a patient who had cleared his HEV infection, indicating that these secreted forms are the main targets of the humoral response during HEV infection.

The level of ORF2intra protein expression and its recognition by MAbs were not affected by mutations of N-glycosylation sites, which is in line with the fact that this protein is not glycosylated^20,21^. However, we showed that ORF2intra proteins displayed both cytoplasmic and nuclear localization, as recently observed in patient liver biopsies^22^. We demonstrated that the ORF2intra protein translocated into the nucleus of HEV producing cells (ORF2ni) independently of its status of N-glycosylation. Altogether, our results demonstrate that, during HEV lifecycle, the ORF2 capsid protein is translocated into the cell nucleus to presumably control certain cellular functions to promote viral replication and/or alter the antiviral response of the infected cell.

We further analyzed the impact of mutations of ORF2 protein N-glycosylation sites on viral RNA production and particle infectivity where none of the mutations had an effect on viral genome replication. In contrast, mutations of N1 glycosylation site inhibited infectivity of particles, mutations of N2 glycosylation site abolished particle assembly and mutations of N3 glycosylation site had little or no effect on assembly of infectious particles. In accordance with Graff *et al*.^14^, whatever the introduced mutation, affecting or not N-glycosylation, the same effects were observed, indicating that N-glycosylation of ORF2 protein does not play any role in assembly of infectious HEV particles. Mutations likely induce modifications in ORF2i protein domains involved in particle assembly. Interestingly, we observed that non-infectious particles were characterized by a slightly lower density, as compared to infectious particles, which might reflect subtle differences in ORF2 assembly leading to non-infectious particles.

It has been suggested that cytoplasmic localization of the ORF2 protein would depend on its ability to be retrotranslocated from the ER by a glycosylation-dependent process^28^. However, the Gly(−) mutant produced ORF2intra protein levels similar to that of the wt ORF2, indicating that ORF2intra proteins are likely not generated from a glycosylation-dependent retrotranslocation process.

Our results demonstrate that ORF2 N-glycosylation is not essential in the HEV lifecycle. However, the ORF2g/c proteins are also modified by O-glycosylation and sialic acids, as recently demonstrated^20,21^. Further studies are now necessary to identify the significance of these modifications in the functionality of ORF2g/c proteins in HEV infection.

In summary, this is the first report on ORF2 N-glycosylation in the physiological context of a gt3 infectious clone, which is the most prevalent genotype in industrialized countries. Our data offer robust and new important findings on the different forms of the ORF2 capsid protein that are likely produced from different pathways^20^ (i) a major non-productive pathway in which ORF2 proteins are addressed to the secretion route where they are glycosylated, maturated and massively secreted, and (ii) a productive pathway in which cytosolic ORF2 proteins are delivered to the virion assembly sites. Finally, we identified a nuclear form of ORF2intra (ORF2ni) suggesting that, during HEV lifecyle, a fine balance of ORF2 partitioning likely occurs between cytosolic, nuclear and reticular pathways.

## Methods

### Chemicals and cell cultures

PLC3^20^ and Huh-7.5^29^ cells were grown in Dulbecco’s modified Eagle’s medium (DMEM) supplemented with 10% inactivated fetal calf serum and 1% of Non-Essential amino acids (Life Technologies) at 37 °C. Transfected PLC3 cells were maintained at 32 °C in a medium containing DMEM/M199 (1 v:1 v), 1 mg/ml of lipid-rich albumin (Albumax I^TM^), 1% of Non-Essential amino acids and 1% of pyruvate sodium (Life Technologies).

### Plasmids and transfection

The plasmid pBlueScript SK(+) carrying the DNA of the full length genome of adapted gt3 Kernow C-1 p6 strain, (GenBank accession number JQ679013, kindly provided by S.U Emerson) was used as a template^19^. The mutants of the ORF2 N-glycosylation sites were generated by site directed mutagenesis. Individual mutations were introduced by sequential PCR steps, as described previously^30^, using the Q5 High-Fidelity 2X Master Mix (New England Biolabs, NEB), then digestions with restriction enzymes and ligation were performed. The double mutants of N-glycosylation sites were generated by exchanging the mutant fragments from single mutants using specific restriction sites. All the mutations were verified by DNA sequencing.

To prepare genomic HEV RNAs (capped RNA), the wild type (wt) and mutant (mt) pBlueScript SK(+) HEV plasmids were linearized at their 3′ end with the MluI restriction enzyme (NEB) and transcribed with the mMESSAGE mMACHINE® kit (Ambion). Capped RNAs were next delivered to PLC3 cells by electroporation using a Gene Pulser Xcell^TM^ apparatus (Bio-Rad)^20^.

### Patient samples

Patient samples were collected in France between 2014 and 2016. Samples were obtained only *via* standard viral diagnostics following a physician’s order (no supplemental or modified sampling). Data were analyzed anonymously. The French Public Health Law (CSP Art L 1121-1.1) does not require written informed consent from patients for such a protocol.

### Kinetic experiments and virus production

PLC3 cells were electroporated with wt and mt HEV RNAs (20 μg/3 × 10^6^ cells). For kinetics experiments, supernatants were harvested 2, 6 and 10 days post-electroporation (d.p.e) and then used for viral titers, RNAs quantification and WB analysis. Transfected cells were lysed 10 d.p.e. in buffer containing 10 mM TrisHCl (pH 7), 150 mM NaCl, 2 mM EDTA, 0.5% Triton X-100, 1 mM PMSF and protease inhibitor cocktail (Complete; Roche). Supernatants and cell lysates were stored at −80 °C until analysis.

### Antibodies

Three mouse anti-HEV ORF2 monoclonal antibodies (MAb) were used: (i) the linear 1E6 MAb (antibody registry #AB-827236, Millipore), (ii) the conformational 4B2 MAb (antibody registry #AB-571018, Millipore) and (iii) the conformational 2E2 MAb (antibody registry #AB-571017, Millipore). Mouse anti-β tubulin (antibody registry #AB-609915) was from Sigma and rabbit anti-Lamin B1 (antibody registry #AB-443298) antibody was from Abcam. Secondary antibodies were purchased from Jackson ImmunoResearch.

### Indirect immunofluorescence

PLC3 cells electroporated with wt and mt HEV RNAs (wt and mt PLC3/HEV cells) were grown on coverslips in 24-well plates and fixed 3 d.p.e. with 3% of Paraformaldehyde (PFA). After 20 minutes (min), cells were washed twice with phosphate-buffered saline (PBS) and permeabilized for 5 min with cold methanol and then with 0.5% Triton X-100 for 30 min. Cells were incubated in PBS containing 10% goat serum for 30 min at room temperature (RT) and stained with the 1E6 MAb for 30 min at RT followed by a Cy3-conjugated goat anti-mouse antibody for 20 min at RT. The nuclei were stained with DAPI (4′,6-diamidino-2-phenylindole). After 2 washes with PBS, coverslips were mounted with Mowiol 4–88 (Calbiochem) on glass slides and analyzed with a LSM 800 confocal laser-scanning microscope (Zeiss) using a x40/1.4 numerical aperture oil immersion lens.

### Quantification of the ORF2 protein nuclear fluorescence

The method was adapted from McCloy *et al*.^31^. Briefly, the ORF2 protein nuclear fluorescence was determined using ImageJ software (version 1.51, NIH). The regions of interest (ROI) were drawn around the nuclei on the immunofluorescence image from PLC3/HEV wt and mt using imageJ ROI tools. Area, integrated density and mean gray values were measured. Then, corrected total cell fluorescence (CTCF) was calculated by the following formula: CTCF = integrated density – (area of selected electroporated cells x mean of background fluorescence around the cells). The exact nuclear fluorescence was = CTCF-the mean of the integrated density of non-infected cells.

### Western blotting analyses

Western blotting analyses were performed as described previously^32^. Briefly, supernatants and lysates of wt and mt PLC3/HEV cells were heated for 20 min at 80 °C in the presence of Laemmli buffer (reducing or non-reducing). Samples were then separated by 10% SDS-PAGE and transferred onto nitrocellulose membranes (Hybond-ECL, Amersham). The targeted proteins were detected with specific antibodies and corresponding peroxidase-conjugated secondary antibodies. The detection of proteins was done by chemiluminescence analysis (ECL, Amersham).

### PNGase-F treatment

Supernatants and lysates of wt and mt PLC3/HEV cells were denaturated for 10 min at 95 °C in glycoprotein denaturing buffer (NEB). Digestions with Peptide-N-Glycosidase F (PNGaseF, NEB) were carried out for 4 h at 37 °C in the presence of 1% NP40 and the buffer provided by the manufacturer (NEB). Samples prepared in the same conditions but without glycosidase were used as controls.

### Nuclear and cytoplasmic extractions

Confluent T75 flasks of wt and mt PLC3/HEV cells (6 × 10^6^ cells) were harvested 12 d.p.e. with trypsin-EDTA. Cells were centrifuged at 4000 rpm for 5 min and washed thrice with PBS. Nuclear and cytoplasmic proteins were extracted using the NE-PER Nuclear and Cytoplasmic extraction Kit (Thermo scientific) following the manufacturer’s recommendations.

### Immunoprecipitations

Polyclonal rabbit anti-mouse antibody (DAKO) was bound to protein A-agarose beads and incubated overnight with mouse anti-ORF2 MAb (1E6, 4B2 or 2E2). Beads were washed thrice with PBS and then incubated for 2 hours at room temperature with supernatants or lysates of wt and mt PLC3/HEV cells. Beads were washed six times with PBS 0.5% NP40 and then heated at 80 °C for 20 min in Laemmli buffer. Proteins were separated by SDS-PAGE and ORF2 proteins were detected by WB using the 1E6 MAb.

### Quantification of the ORF2 protein levels by ELISA

The supernatant of wt and mt PLC3/HEV cells and non-transfected PLC3 cells (CTL) were diluted in PBS (1:250 and 1:500). HEV ORF2 Ag levels were measured with the Wantaï HEV-Ag ELISA^Plus^ kit (Wantaï Biological Pharmacy Enterprise), as recommended by the manufacturer.

### Intracellular viral particles and RNAs

The procedure was adapted from Emerson *et al*.^33^. Briefly, confluent T25 flasks of wt and mt PLC3/HEV cells were trypsinized and cells were centrifuged for 10 min at 1500 rpm. Cells were washed thrice with PBS. Intracellular viral particles were extracted by resuspending cells in 1 ml of sterile MilliQ water at room temperature. Cells were vortexed vigorously for 20 min and then 110 µl of sterile 10X PBS were added. Samples were clarified by centrifugation 2 min at 14000 rpm. The supernatants containing intracellular particles and RNAs were collected and stored at −80 °C until analysis.

### HEV RNAs extraction and quantification

Supernatants collected at 2, 6 and 10 d.p.e and intracellular viral particles produced in wt and mt PLC3/HEV cells were submitted to viral RNAs extraction. HEV RNA levels were quantified by RT-qPCR, as described previously^34,35^.

### Infectious titers

Huh 7.5 cells (3 × 10^3^) seeded in 96-well plates the day before were infected with serial dilutions of supernatants or intracellular viral particles from wt and mt PLC3/HEV cells. Three days post-infection, cells were fixed and processed for indirect immunofluorescence. Cells labeled with anti-ORF2 antibody 1E6 were counted as infected cells. The number of infected cells was determined for each dilution and used to define the infectious titers in focus forming unit (FFU)/ml.

### Mass spectrometry

N-terminus identification of the ORF2c protein was performed as in Montpellier *et al*.^20^. For N-glycans analyses, ORF2g/ORF2c proteins were immunoprecipitated with the 4B2 anti-ORF2 antibody and denaturated for 10 min at 95 °C in glycoprotein denaturing buffer (NEB). Proteins were treated or not with PNGaseF^20^, as described above, and resolved by 10% SDS-PAGE. Colloïdal blue stained bands corresponding to ORF2g and ORF2c proteins in WB were cut into two slices for in-gel digestion with trypsin or AspN. NanoLC-MSMS analyses of the protein digests were performed as described in Montpellier *et al*.^20^

MS/MS data was interpreted using search engine Mascot (version 2.4.0, Matrix Science) with a tolerance on mass measurement of 10 ppm for precursor and 0.02 Da for fragment ions, against a composite targetdecoy database (40584 total entries) built with the sequences of ORF2 (H9E9C9_HEV) and the PNGaseF-deglycosylated ORF2 protein in which the three N-glycosylated sites were replaced by Asp(D) residues, fused with a Swissprot homo sapiens database (TaxID = 9606, 20 May 2016, 20209 entries) and a list of classical contaminants (119 entries). Carbamidomethylation of cysteine residues, oxidation of methionine residues and protein N-terminal acetylation were searched as variable modifications. Up to three trypsin or Asp-N missed cleavage were allowed. Semi-specific cleavage was also authorized. Spectral counting was performed without MS score filtering. Peptides quantitation of ORF2 and PNGaseF-deglycosylated ORF2 protein was performed on MS1 level using Skyline (ver. 3.7)^36^. After automated peak picking and retention time alignment of Skyline, a manual correction of wrong peak boundaries was performed and normalized total areas of peptide peaks were exported.

### Density gradients

Density gradients on supernatants of wt and mt PLC3/HEV cells were performed as described in Montpellier *et al*.^20^.

## Supplementary information

Supplementary Information
