# Supplementary material for: New insights into the ORF2 capsid protein, a key player of the hepatitis E virus lifecycle
Source: Sci Rep. 2019 Apr 18;9:6243. doi: 10.1038/s41598-019-42737-2 (PMC6472401; doi:10.1038/s41598-019-42737-2)
Supplement: Supplementary file 1 — Supplementary Information [file 41598_2019_42737_MOESM1_ESM.pdf]

SUPPLEMENTARY INFORMATION

New insights into the ORF2 capsid protein, a key player of the hepatitis E virus lifecycle

Maliki Ankavay, Claire Montpellier, Ibrahim M. Sayed, Jean-Michel Saliou, Czeslaw Wychowski, Laure Saas, Sandrine Duvet, Cécile-Marie Aliouat-Denis, Rayan Farhat, Valentin de Masson d'Autume, Philip Meuleman, Jean Dubuisson and Laurence Cocquerel

Supplementary Figure 1: Identification of the N-terminus of the ORF2c protein. ORF2c proteins were immunoprecipitated with an anti-ORF2 antibody (4B2), denaturated and incubated or not with N-succinimidyloxycarbonylmethyl tris (2,4,6-trimethoxyphenyl) phosphonium bromide (TMPP), which binds specifically to the N-terminus of intact proteins. Proteins were resolved by SDS-PAGE, digested in-gel with trypsin or AspN and analyzed by nanoLC-MS/MS. (A) Peptide covering is highlighted in grey on the sequence. Ser^{102} in bold corresponds to the first aa of ORF2c that was identified by TMPP labeling. (B) and (C) MS/MS spectrum of N-terminal peptides of the ORF2c protein. (B) Tryptic peptide obtained from TMPP-labeled ORF2c protein. (C) AspN peptide obtained from TMPP-labeled ORF2c protein. +572 corresponds to the TMPP mass increment following TMPP labeling.

Supplementary Figure 2: Specificity of the anti-ORF2 1E6 antibody. (a) Detection of ORF2 protein by WB using the 1E6 antibody on supernatants and cell extracts of PLC3/HEV cells and non-transfected PLC3 cells. (**b**) Uncut gels are displayed.

Supplementary Figure 3: Characterization of wt and mutant ORF2 proteins by western blot (WB). For each panel showing western-blotting results, uncut gels are displayed.

Supplementary Figure 4: Impact of mutations of ORF2 protein N-glycosylation sites on antibody recognition. For each panel showing western-blotting results, uncut gels are displayed.

Supplementary Figure 5: Impact of mutations of N-glycosylation sites on ORF2 protein nuclear localization. For each panel showing western-blotting results, uncut gels are displayed.

Supplementary Figure 6: Impact of mutations of ORF2 protein N-glycosylation sites on particle density. For each panel showing western-blotting results, uncut gels are displayed.

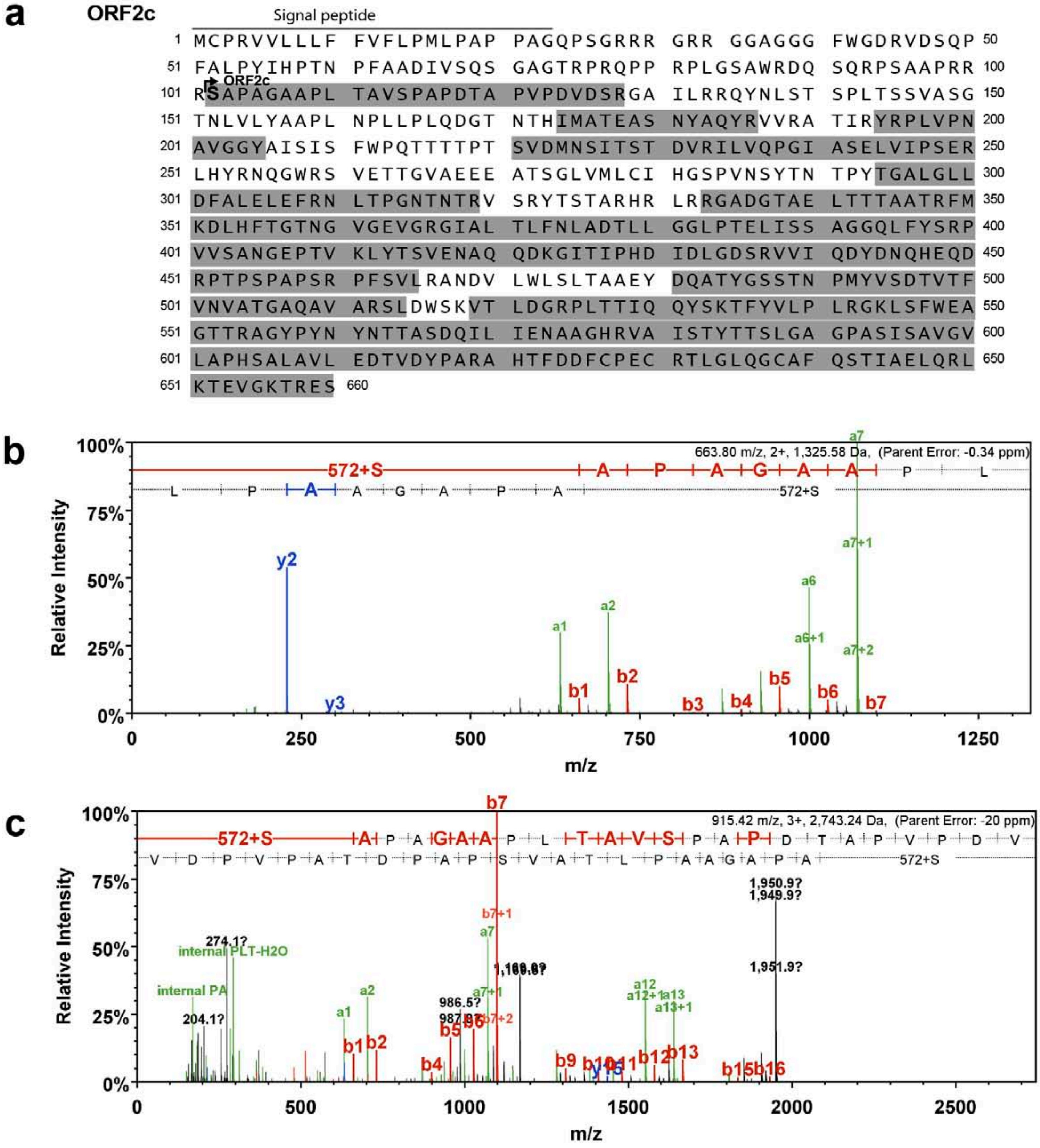

Supplementary Figure 1

a

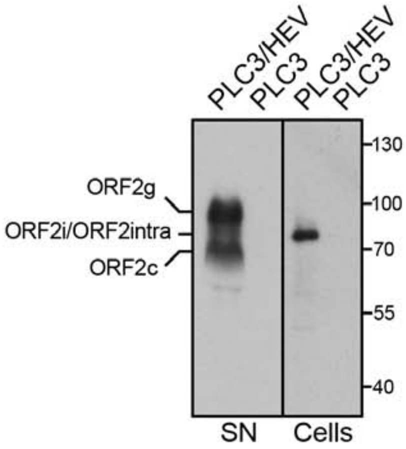

b

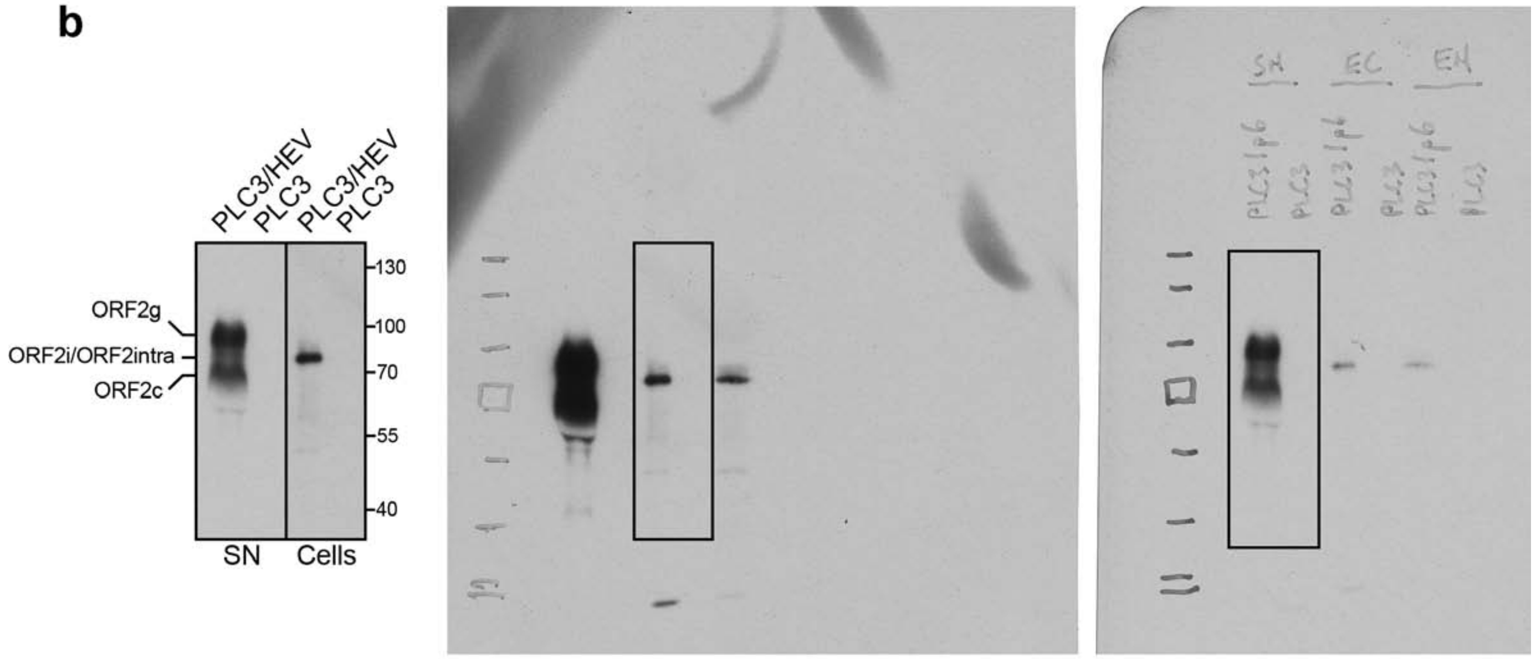

Supplementary Figure 2

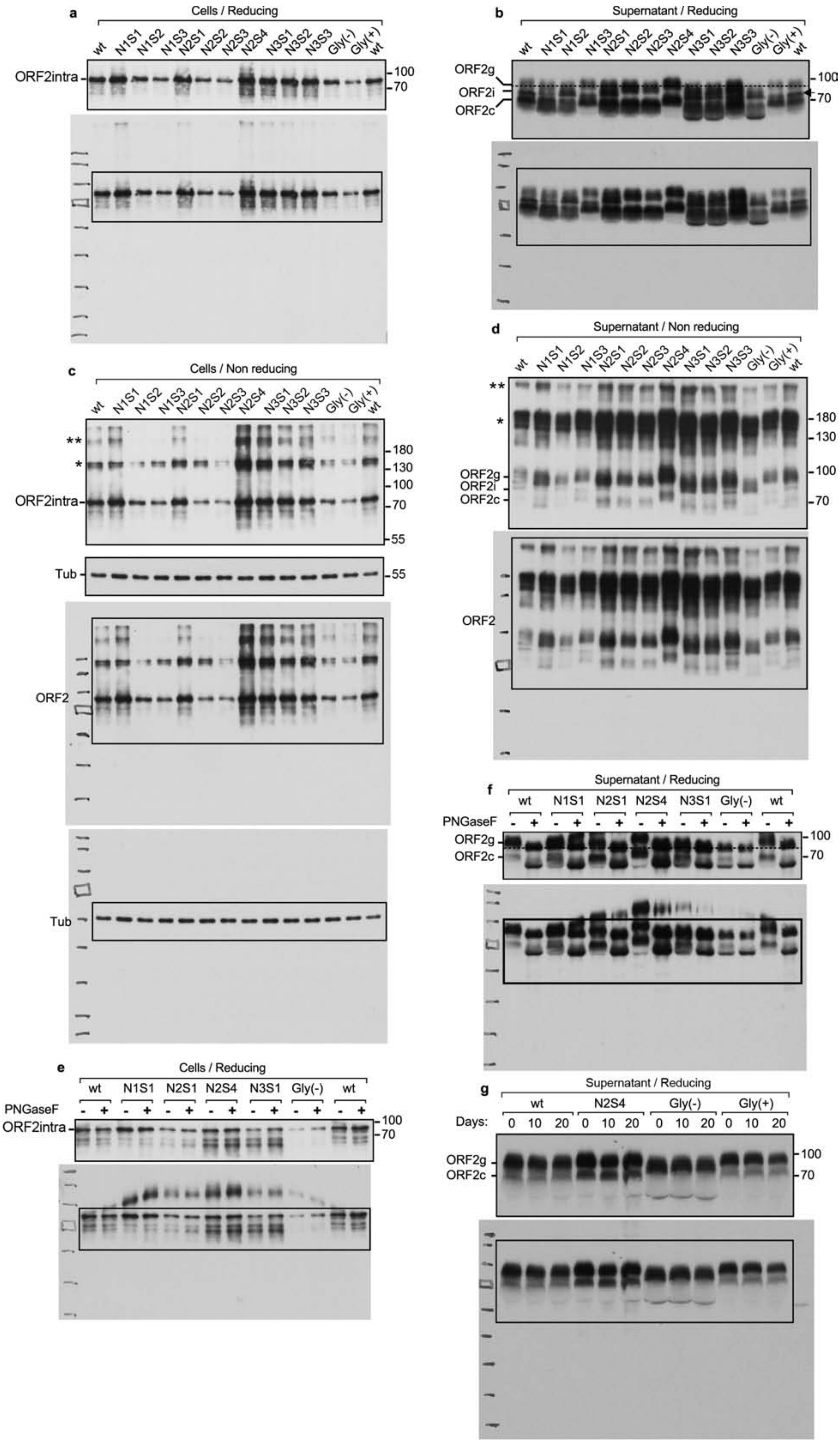

Supplementary Figure 3

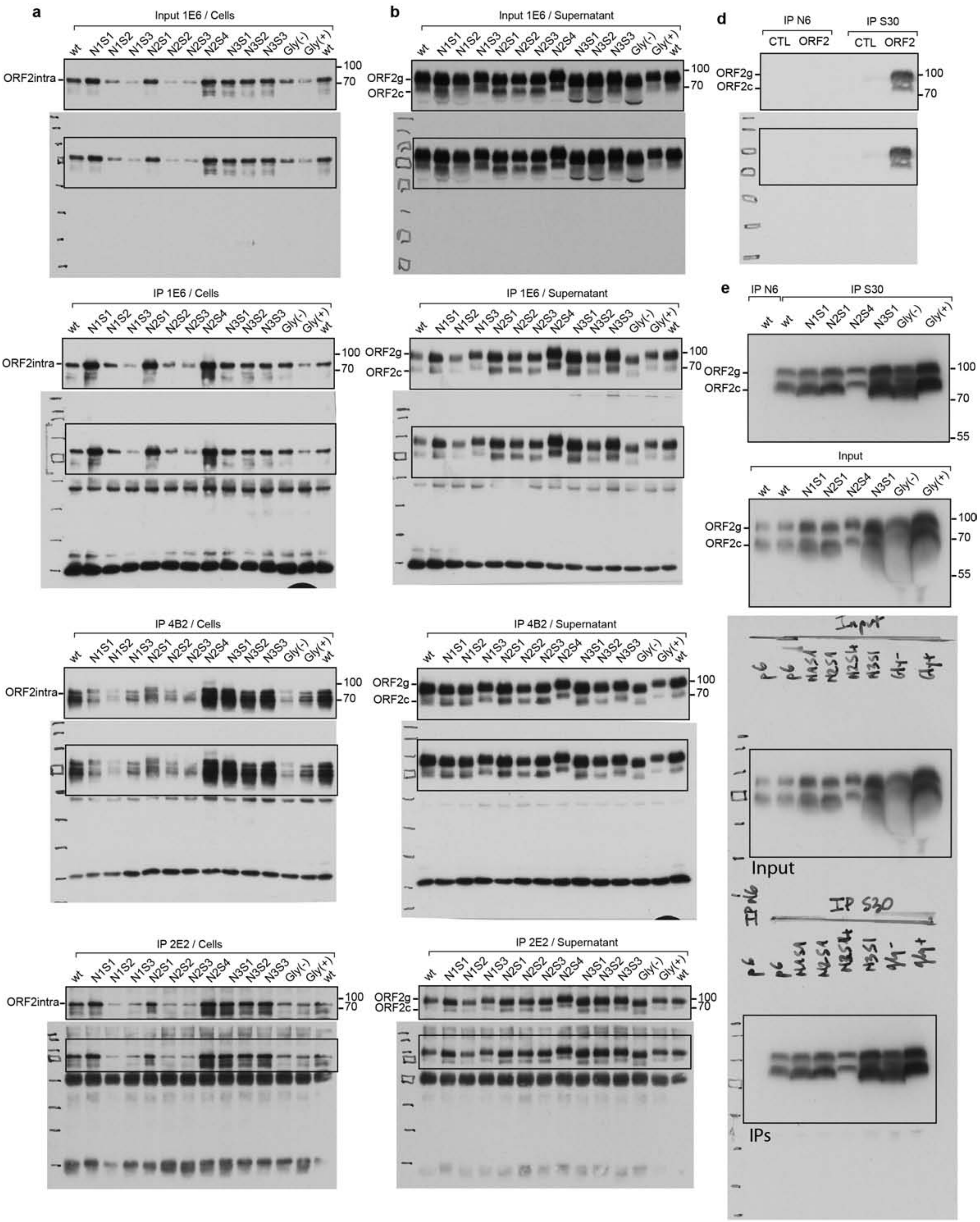

Supplementary Figure 4

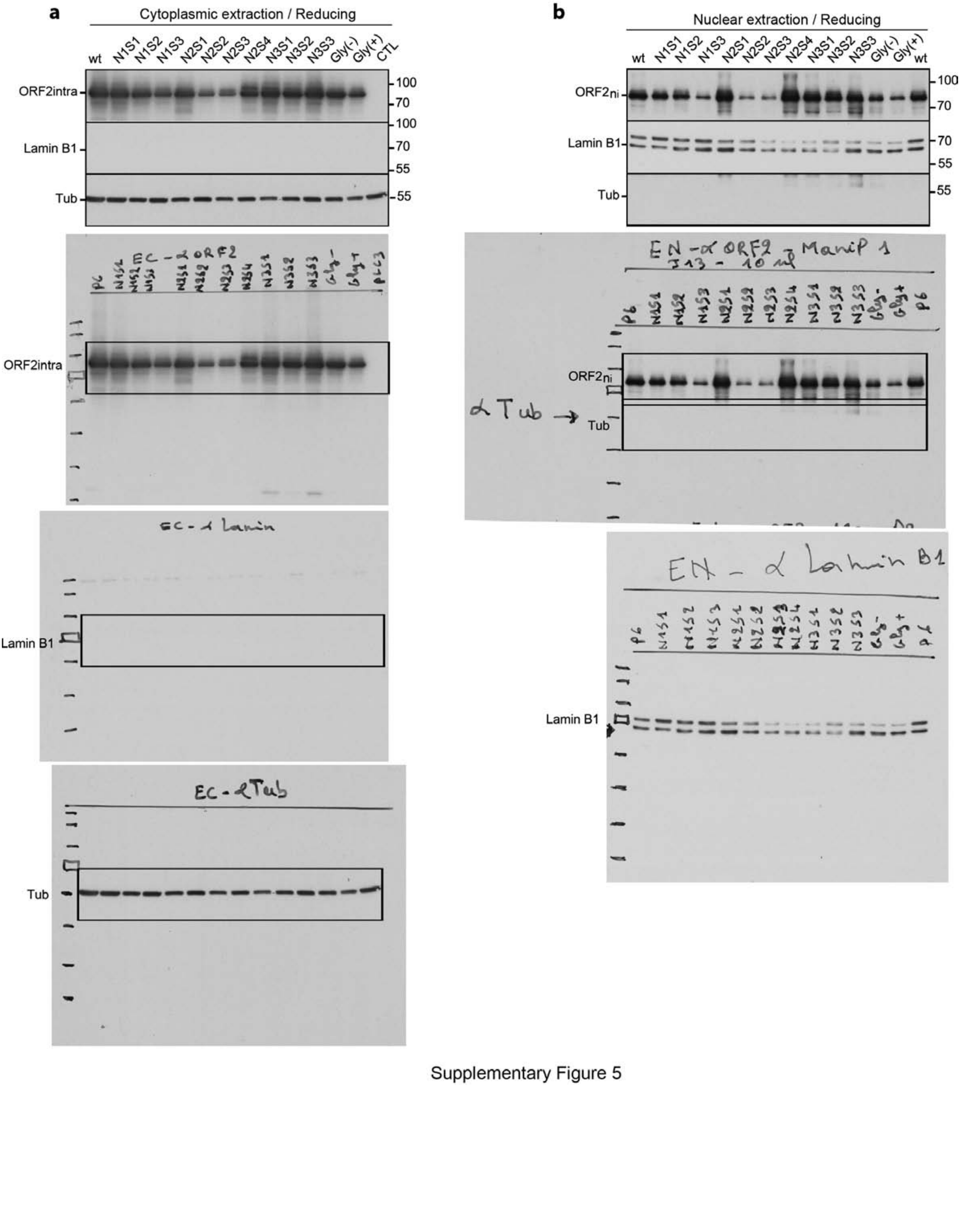

Supplementary Figure 5

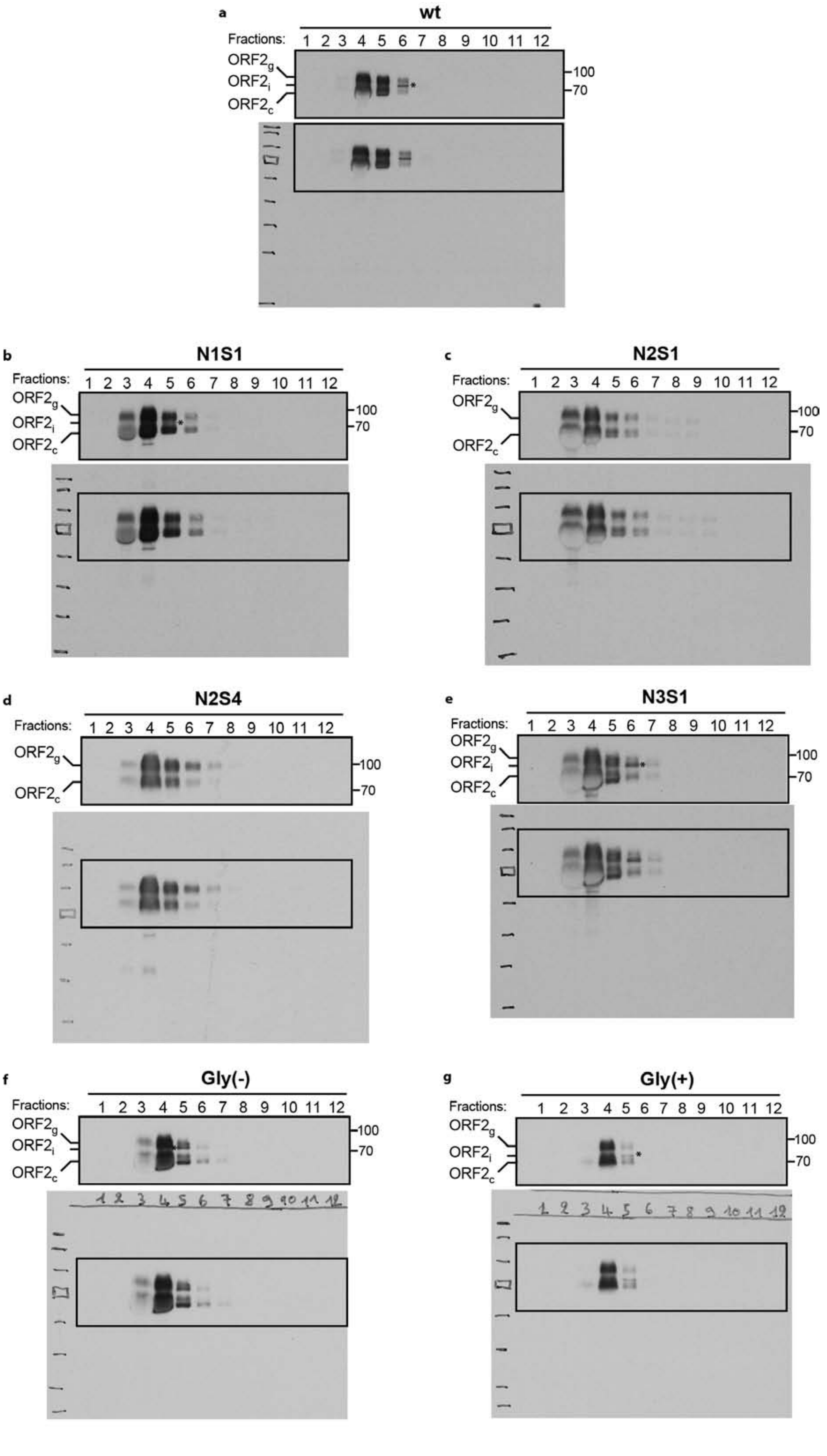

Supplementary Figure 6
